# Feather macrostructure is marginally correlated with temperature range but not urbanization across California

**DOI:** 10.1038/s41598-025-04378-6

**Published:** 2025-07-07

**Authors:** Wilmer Amaya-Mejia, Sara Lim, Lillian Ma, Allison J. Shultz, Pamela Yeh

**Affiliations:** 1 Ecology and Evolutionary Biology Department, University of California, Los Angeles, Los Angeles, CA USA; 2 Ornithology Department, Natural History Museum of Los Angeles County, Los Angeles, CA USA; 3 Santa Fe Institute, Santa Fe, NM USA

**Keywords:** Avian, Cities, Dark-eyed junco, Plumage, Heat-island effect, Climate change, Climate-change ecology, Urban ecology, Ecology, Zoology

## Abstract

Urban environments are often associated with resource and environmental differences providing potential novel selection pressures compared to adjacent unmodified landscapes. While these characteristics (e.g., heat islands, reduced vegetation) can contribute to differences in certain behaviors, morphology, or physiological traits, there is mixed evidence on how and to what extent populations are responding. In this study, we compared the feather morphology of Dark-eyed Junco (*Junco hyemalis*) populations established across an urbanization gradient. We examined whether differential temperature regimes, related to urbanization, correspond with significant variations to the proportion of down. We sampled ventral and dorsal feathers from 256 individuals throughout central and southern California at varying degrees of urbanization. Dorsal feathers had a higher proportion of down compared to ventral feathers, but did not differ between populations. Urbanization did not significantly correlate with feather morphology. Ventral feathers had a greater proportion of down as the range of temperature increased, but this correlation was marginal. Our results show that despite urbanization altering fine-scale habitat conditions, these did not correspond with rapid feather morphological variations. Whether this is the case for other feather types or across species is still unknown but would provide insight into the complex effects of urbanization on wildlife biology.

## Introduction

Urbanization is a multifaceted phenomenon that encompasses a number of characteristics including localized heat-island effects^1,2^, increased impermeable surface coverage, reduced vegetation^3^, and increased human presence^4,5^. This plethora of environmental changes has resulted in variable responses by wild avian populations. While there are instances of plasticity in response to urban-associated habitat changes (e.g., decreased human-avoidance behaviors^6,7^, ground nesters building off-ground nests^8,9^, shifting songs due to urban acoustics^10,11^), phenotypic variations can be conditional (e.g., age-specific differences in body condition^12^ and feather morphology^13,14^) and even inconsistent between populations^15–17^. This degree of complexity in how traits respond to urbanization highlights the need for additional studies, ideally across multiple sites varying in degree of urbanization, to fully characterize phenotypic variations.

The urban heat island (UHI) effect is one attribute of cities which may contribute to phenotypic variation^1–3,18^. Generally, urban centers are associated with higher ambient temperatures compared to nearby non-urban habitats^1,3^. Differences in ambient temperature can select for adaptations related to thermoregulation, as suggested by studies that compared to birds across a climate gradient, birds sampled from areas with colder ambient temperatures had feather morphology consistent with increased insultation, more downy barbs, and more downy plumage overall^19–23^. Therefore, the UHI effect could represent a novel source of temperature-associated selection pressures. However, the UHI effect is heterogenous^2^ and greenspaces within cities create climate refugia^1–3,18^. These climate refugia, in combination with behavioral adaptations^24^ and morphological trait plasticity^25,26^, may assist in offsetting UHI effects and ease selection pressures. By characterizing thermoregulatory traits, such as feather morphology, from populations found across different cities, we can continue to examine the degree to which urbanization impacts local wildlife.

Feathers are defining traits of birds and serve as a major interface between a bird and its environment^27^. In addition to the aforementioned thermoregulatory functions^21,28^, feather functions can include resistance to UV radiation^29^, heavy metal sequestration^30,31^, water repellence^20^, among others^27,32^. Contour feathers comprise the majority of a bird’s plumage and serve as a primary interface with the environment^19^. Depending on the environmental conditions, such as those presented by a climate gradient, feather structural traits may vary; for example, contour feathers may be longer and have a larger proportion of down to increase the amount of air trapped along the body and improve heat retention in cold environments^19,20,25^. Despite this preexisting body of work examining contour feather structural trait variation, there is still a need to examine how *recent* environmental changes, such as those posed by urbanization, can affect feather morphology and function and whether this has a significance for avian biology. As ambient temperature increases, linked to UHI effects, the thermoregulatory demands experienced by urban birds could change and reduce the need for feather morphology related to insulation^1,2,33^.

The plumulaceous portion of contour feathers (proportion of down) is directly associated with heat retention^20^ and is therefore more likely to respond to changes in ambient temperatures. The lengths and proportions of the feather composed of the pennaceous barbs (those more structurally rigid and that generally contain the structures of pigments that produce the visible color) and the downy barbs (those that are often looser and are primarily associated with heat retention) varies across life histories and environmental gradients^19,20,23,34^. The barbs (branching structures) in the downy region of the feather create air pockets that retain body heat close to the bird’s body, reducing radiative loss to < 5%^33^. The combination of physical structures of feathers and a few notable behavioral traits, such as ptiloerection and shivering^24^, result in improved cold tolerance without necessarily detracting from heat tolerance^19^. Hence, local climatic conditions have been linked to phenotypic variability of feather morphology^19,20,22,35^. Further, in the face of rapidly changing habitat conditions, studies suggest that plasticity can contribute to rapid shifts in feather morphology^25,26,36^. However, it is unknown whether any changes that arise are consistent across different populations. To address this, our study examines whether the increased ambient temperatures produced by UHIs correspond with a reduced proportion of down in contour feathers of Dark-eyed Juncos (*Junco hyemalis*).

Here, we explore whether urbanization and UHIs correlate with contour feather morphological variation (proportion of down) of the Dark-eyed Junco in California, USA. The Dark-eyed Junco is a species with urban and non-urban populations that are showing rapid evolution in response to urban environments, making it a model species for studying the effects of urbanization^8,16,37–39^. Previous studies of trait variation between urban and non-urban populations have primarily focused on behaviors^6,8,11,37,40^, with only a few morphological studies (e.g., sexually selected tail feather morphology^17,38^ and bill and wing morphology^16^). Here, we investigate whether the proportion of down shows variation across such fine scales, and specifically whether or not this variation is associated with urbanization and related UHI effects. Specifically, birds in colder environments have been found to have a larger proportions of down to facilitate heat retention^19,20,25^. Therefore, we hypothesize that the UHI effect, characterized by stable, warm ambient temperatures, will reduce selective pressures for insultation, resulting in a reduction in the proportion of down when comparing contour feathers in urban birds to non-urban birds. However, as the UHI effect constitutes a relatively recent, fine-scale phenomenon that can be offset by localized climate refugia, we predict we will observe a weak, negative correlation between ambient temperature and proportion of down. Notably, these results are limited to the breeding period of the Dark-eyed Junco and may not reflect prolonged differences.

## Methods

### Experimental model

We captured 256 Dark-eyed Juncos across central and southern California during the 2022 (n = 47) and 2023 (n = 209) breeding season (February–August) between 07:00 and 12:00. We mist netted at six locations on average per day, and at each location, the net was open for 30 min during which playbacks were used to attract juncos in the area. Playbacks consisted of singing juncos from Los Angeles. We color-banded each junco with a unique three color band and one USGS aluminum band combination and recorded various metrics as part of a long-term study. Metrics included bill dimensions (width, depth, and length), body weight, wing chord length, tail length, and tarsus length^16^. We quantified sex differences based on presence of brood patch or cloacal protuberance, as well as plumage characteristics. The juncos were aged as after second year (ASY), second year (SY), after hatch year (AHY), or hatch year (HY) based on known plumage and molt limit^41^. Data from HYs and recaptured juncos were excluded from this study.

**Fig. 1:**
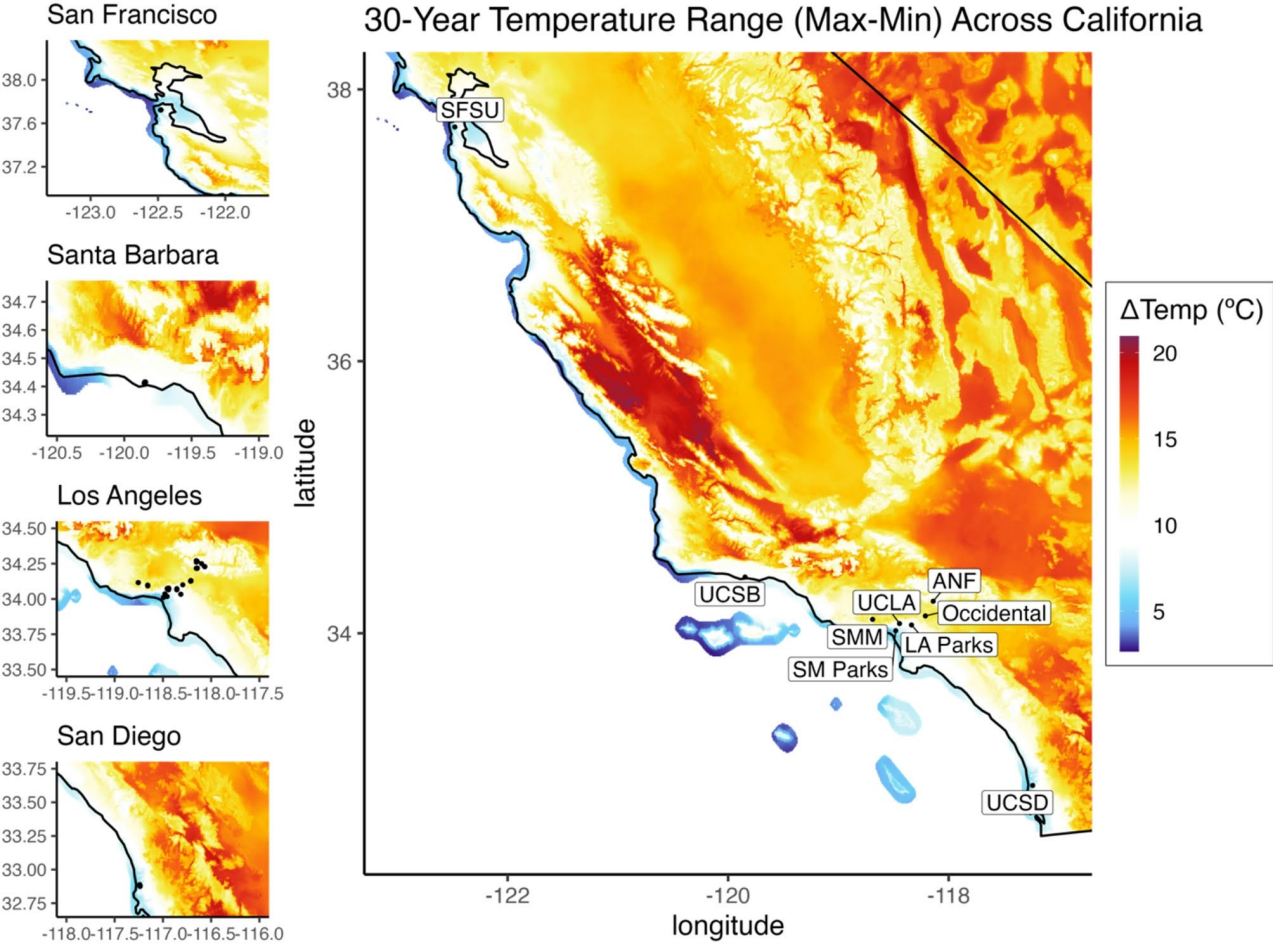
Temperature range (max–min) averaged over the past 30 years (1991–2020) across California. Large map (right) shows the temperature range across central and southern California with the study sites labelled. Smaller maps (left) show the counties (San Francisco, Santa Barbara, Los Angeles, and San Diego) within which our study sites are found, and coordinates for each Dark-eyed Junco sampled are represented by the black points. Temperatures with a larger range are represented by dark red, and temperatures with a smaller range are represented by dark blue.

We collected 2–6 contour feathers from male and female adult juncos in definitive basic plumage. To sample feathers, banders removed 1–3 feathers from the anterior region of the mantle (dorsal) and 1–3 feathers from the anterior region of the breast (ventral) of each bird. Feathers were stored dry in envelopes until processing. Our sites consist of the University of California, Los Angeles (UCLA, n = 121), the University of California, San Diego (UCSD, n = 22), the University of California, Santa Barbara (UCSB, n = 18), Occidental College (Occ, n = 22), San Francisco State University (SFSU, n = 6), Santa Monica Parks (SM Parks, n = 9), Los Angeles Parks (LA Parks, n = 10), as well as nearby mountain regions in the Santa Monica Mountains (SMM, n = 9), and the Angeles National Forest (ANF, n = 35; Fig. 1). The minimum distance between our sampling sites was 4700 m (between UCLA and SM Parks). This distance is further than the expected maximum distance of 174 m that a junco will fly during the breeding season^42^, reducing the possibility of the same individual moving between sites. Coordinates of each banding site were recorded and used to obtain corresponding environmental and climatic conditions.

All methods were carried out in accordance with relevant ARRIVE methods required for observational animal research^43^ and in accordance with institutional guidelines and regulations. Animal handling in this study adhered to protocols approved by the Institutional Animal Care and Use Committee (IACUC) of UCLA (ARC-2018-007-AM-004). Banding efforts were conducted in compliance with the Ethics and Responsibilities of Bird Banders published by the US Geological Survey Federal Bird Banding Laboratory (Permit #23809) and as outlined by the State of California Department of Fish and Wildlife Scientific Collecting Permit—Specific Use (S-191300002-20288-001-02) for taking/possession of wildlife for scientific purposes.

### Macrostructure measurements

We calculated the proportion of the feather surface area that consists of plumulaceous barbs (henceforth “proportion of down”) and then averaged these values for each individual bird. To determine the proportion of down, we scanned each feather using a CanoScan LiDE 400 Color Image Scanner at a resolution of 600 dots per inch (dpi). When scanning the images, we placed the feathers on a transparency sheet labeled with a 10 mm scale and weighed each feather down with a 0.13–0.19 mm thick coverslip. A colored sheet of cardstock laid on top maximized the contrast between the desired region and the background.

**Fig. 2:**
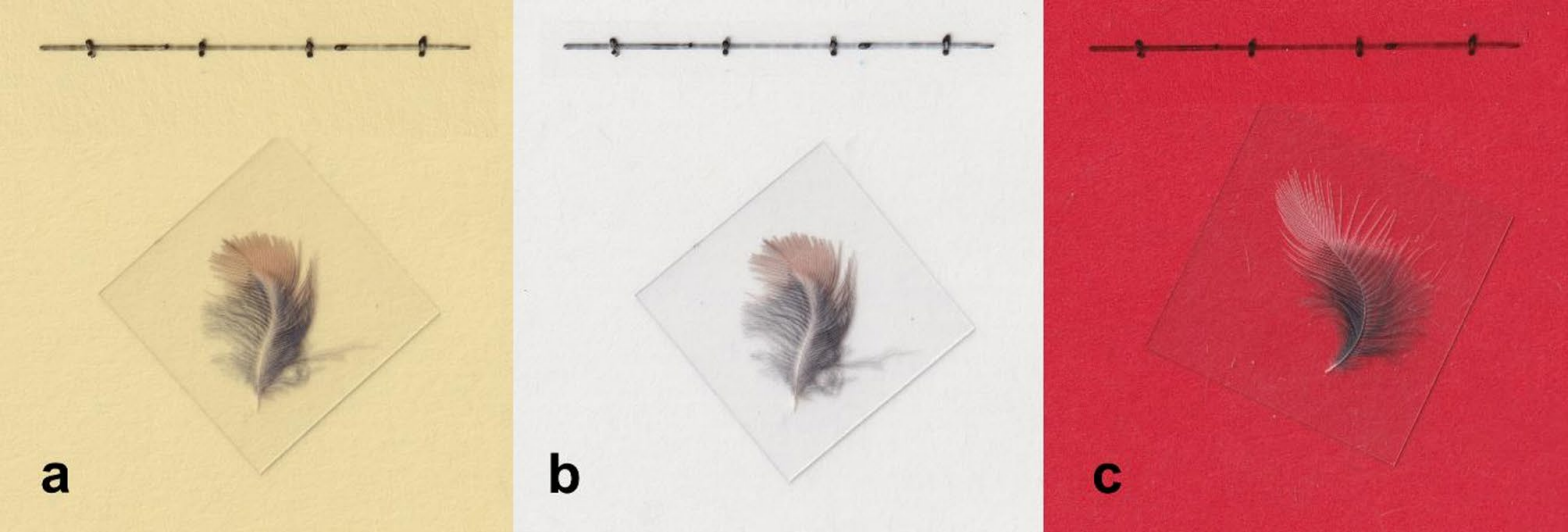
Scanned feathers used to quantify feather morphology. Backgrounds included to ensure color thresholds successfully capture the desired region. Dorsal feathers were placed against a light yellow background to capture presence of pennaceous structures (**a**) or against a white background to capture the entire feather surface area (**b**). Red background facilitated the differentiation between regions with downy area and the entire feather surface area of ventral feathers (**c**).

To measure the relative proportion of down, we developed color thresholds using a hue-saturation-brightness color space in ImageJ for the ventral and dorsal feathers in order to increase repeatability and reduce potential observer bias^44^. For dorsal feathers, a yellow (#ebd798) background and a white (#e8e7e5) background provided the best contrast to measure the pennaceous region and total surface area, respectively (Fig. 2a, b). To analyze ventral feathers, we measured the proportion of down and total surface area against a red (#c71934) background (Fig. 2c). We then selected and measured the area above the set color threshold. To calculate the area of down in dorsal feathers, we subtracted the area of pennaceous barbs from the total area. We then calculated the proportion of down for dorsal and ventral feathers by dividing the area of down by total surface area.

Using a subset of feathers, our values correlated with previously published metrics used to measure the proportion of down and pennaceous feathers based on a Kendall’s rank correlation tau, where the color difference measures of surface area were strongly correlated with the length of down/pennaceous regions along the shaft of the feathers (τ = 0.388, *p* < 0.001, n = 100)^23,34,35,45^. We selected our metric to reduce potential observer bias when quantifying feather barbs.

We assessed repeatability of our measurements with feathers from museum specimens to reduce stress on living birds. Repeatability of our measurements was included to account for sampling bias^23,34,46,47^. Museum specimens provided by the Occidental College Moore Laboratory of Zoology were all collected within five years of our study from Los Angeles (Specimen numbers: MLZ:Bird:70290; MLZ:Bird:70291; MLZ:Bird:70222; MLZ:Bird:70246; MLZ:Bird:70221; MLZ:Bird:70292). We randomly sampled a different number of feathers from each specimen (n = 5, 6, 8, 9, 12, 15) and calculated the proportion of down using the methods previously described. We then ran a linear model for variance of the proportion of down of dorsal feathers against sample size, defined as the number of feathers sampled from a given specimen. We repeated this model for ventral feathers.

### Categorization of environmental conditions

Temperature data from the PRISM Climate Group was available using the *prism* 0.2.0 package in R^48^. Our climatic variables include mean temperature (T_mean_: μ = 17.3 ºC, SD = 1.1, range = 13.4 to 18.9, CV = 6.28%) and the temperature range, based on the difference between the maximum and minimum temperatures (ΔTemp: μ = 9.9 ºC, SD = 1.7, range = 6.1 to 13.6, CV = 16.90%; Fig. 1). For each variable, we extracted averages over 30 years (1991–2020) at a resolution of 800 m using the *terra* 1.7^49^, *sp* 2.0^50^, and *sf* 1.0^51^ packages. Selected values capture the broadest conditions but may not accurately represent the strongest selective pressures. Temperature extremes, for instance, could be more impactful but were not available for our study.

Measurements of urbanization are based on Built-Up index (BU) calculated using raster images from Collection 2 Level 2 LandSat 8 data available from the U.S. Geological Survey Earth Resources Observation and Science (EROS) Center Science Processing Architecture (ESPA). These raster images are at a 30 m resolution within one month of the sampling date where cloud cover was less than 8%. We used QGIS^52^ to calculate BU index with the following equation:

$$\begin{aligned} & Built{ - }Up = Normalized\;Difference\;Built{ - }up\;Index\; \left( {NDBI} \right) \\ & \quad - Normalized\; Difference\; Vegetation \;Index\; \left( {NDVI} \right) \\ \end{aligned}$$

$$NDBI = \frac{SWIR1 - NIR}{{SWIR1 + NIR}},\;NDVI = \frac{{NIR {-} Red}}{NIR + Red}$$

At each sampling coordinate, we then averaged the BU index within a 50 m radius (μ = −0.236, SD = 0.095, range = −0.531 to −0.041, CV = 40.33%), which represents the average territory size of Dark-eyed Juncos recorded by previous literature as well as our own personal observations^42^. This method of averaging BU index is most accurately able to capture the degree of urbanization of our study sites^53^. Detailed information on the BU, T_mean_, and ΔTemp for different sites is provided (Supplemental Table S1).

### Quantification and statistical analysis

We analyzed our data using R 4.3.0^54^ and created figures using the package *ggplot2* 3.4.2^55^*.* We determined appropriate statistical analysis based on whether the data was normally distributed using the Shapiro–Wilk Normality Test. The residuals for the final models were reviewed to confirm that appropriate assumptions are met. Statistical significance was set as α = 0.05. All p-values were reported unless they were less than 0.001.

We quantified urban heat island effects for our study sites. Our data followed a non-normal distribution (Shapiro–Wilk: T_mean_: *p* < 0.001, n = 256; ΔTemp: *p* < 0.001, n = 256; BU: *p* < 0.001, n = 256), so we used Kendall’s rank correlation tau to assess for a correlation between temperature and urbanization variables and to quantify UHI effects.

To compare the proportions of down across our nine sites, we conducted an ANOVA test for the normally distributed dorsal feathers (Shapiro-Wilks: *p* = 0.174, n = 252) and a Kruskal–Wallis test for the non-normally distributed ventral feathers (Shapiro–Wilks: *p* < 0.001, n = 256). We then ran a Wilcoxon signed rank exact test to compare the means and an F-test to compare the variances in order to assess the differences in proportions between dorsal and ventral feathers.

To determine if feather macrostructure varied in response to either temperature or BU, we built models and ranked them according to lowest small-sample corrected Akaike information criterion (AICc) using the AICc function in the *MuMIn* package^56^. There was no significant difference between sex (Wilcoxon rank sum test: dorsal, *p* = 0.073, n_M_ = 181, n_F_ = 69; ventral, *p* = 0.562, n_M_ = 185, n_F_ = 69), so sex was not included in subsequent model selections. Additionally, the ICC of the random effect of site was low for models of both dorsal (ICC = 0.009) and ventral (ICC = 0.106) feathers, so we opted against including site as a random effect. For the dorsal feathers, we removed any outliers (|z|> 3) and found that the data followed a normal distribution, so we ran linear models on the subset of data. Since the proportion of down in ventral feathers did not follow a normal distribution, we chose to run generalized linear models on all the ventral feathers using an inverse Gaussian distribution, chosen based on examination of residuals. For the two feather types, we had three models based on our three predictor variables: (1) BU, (2) ΔTemp, and (3) the null model. Model outputs including estimates, standard error*, p*-value, AICc, ΔAICc, and AICc weights were reported in Table 1.

**Table 1:** Ranked models of BU is the Built-Up Index. ΔTemp (ºC) is the temperature range (max–min) from 1991–2020.

Response	Model	df	Estimate (Intercept)	*p*-value	AICc	ΔAICc	AICc weight
Ventral	~ ΔTemp	3	-0.068 ± 0.028 (3.164 ± 0.284)	0.016* (< 0.001***)	-483.66	0	0.840
	Null	2	(2.489 ± 0.049)	(< 0.001***)	-479.69	3.97	0.115
	~ BU	3	0.181 ± 0.509 (2.532 ± 0.130)	0.723 (< 0.001***)	-477.77	5.89	0.044
Dorsal	Null	2	(0.736 ± 0.004)	(< 0.001***)	-734.48	0	0.953
	~ ΔTemp	3	0.002 ± 0.002 (0.718 ± 0.021)	0.398 (< 0.001***)	-727.35	7.12	0.027
	~ BU	3	-0.012 ± 0.037 (0.732 ± 0.009)	0.75 (< 0.001***)	-726.73	7.75	0.020

Models for each response are ranked from most to least supported based on lowest AICc score. Ventral responses are modeled by generalized linear models with an inverse Gaussian distribution. Dorsal responses are modeled by linear model with a Gaussian distribution. Estimate reported as β ± se. ***p < 0.001, **p < 0.01 and *p < 0.05.

## Results

Study sites ranged from highly urban to moderately vegetated (BU: μ = −0.236, SD = 0.095, range = −0.531 to -0.041) with moderate temperatures (T_mean_: μ = 17.3 ºC, SD = 1.1 ºC, range = 13.4 ºC to 18.9 ºC; ΔTemp: μ = 9.9 ºC, SD = 1.7 ºC, range = 6.1 ºC to 13.6 ºC). Within these sites, urbanization was more variable (BU: CV = 40.33%) than temperatures (T_mean_: CV = 6.28%; ΔTemp: CV = 16.90%). There was no correlation between the BU and T_mean_ (Kendall’s rank correlation tau: τ = −0.019, *p* = 0.668, n = 256). There was a negative correlation between BU and ΔTemp, where urban sites had more stable temperatures than non-urban sites (Kendall’s rank correlation tau: τ = −0.280, *p* < 0.001, n = 256).

**Fig. 3:**
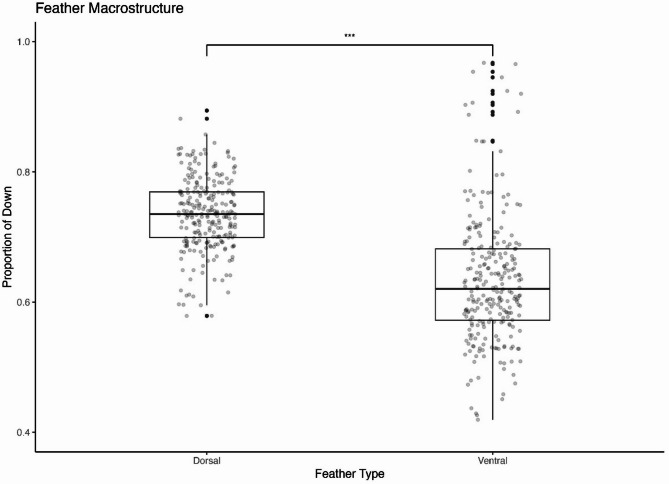
Feather macrostructure differs between dorsal and ventral feathers. Light grey points represent all individual juncos, and black points represent outliers. The proportion of down observed in dorsal feathers is significantly higher (***p < 0.001) and less variable compared to ventral feathers.

The proportion of down did not differ among the nine study sites for either dorsal feathers (ANOVA: F = 1.177, df = 8, *p* = 0.314) or ventral feathers (Kruskal–Wallis: X^2^ = 15.499, df = 8, *p* = 0.050). Dorsal feathers (μ = 0.736, SD = 0.056, range = 0.579 to 0.894, CV = 8%, n = 252) had a significantly greater proportion of down compared to ventral feathers (μ = 0.634, SD = 0.099, range = 0.419 to 0.967, CV = 16%, n = 256) and reflects a large effect of body region on feather morphology, based on a Wilcoxon rank sum test (W = 54,284, *p* < 0.001, *r* = 0.59; Fig. 3). The dorsal feathers also had a lower variance in their proportion of down than ventral feathers (F = 0.321, *p* < 0.001, 95% CI = 0.26—0.41, df_dorsal_ = 251, df_ventral_ = 255).

**Fig. 4:**
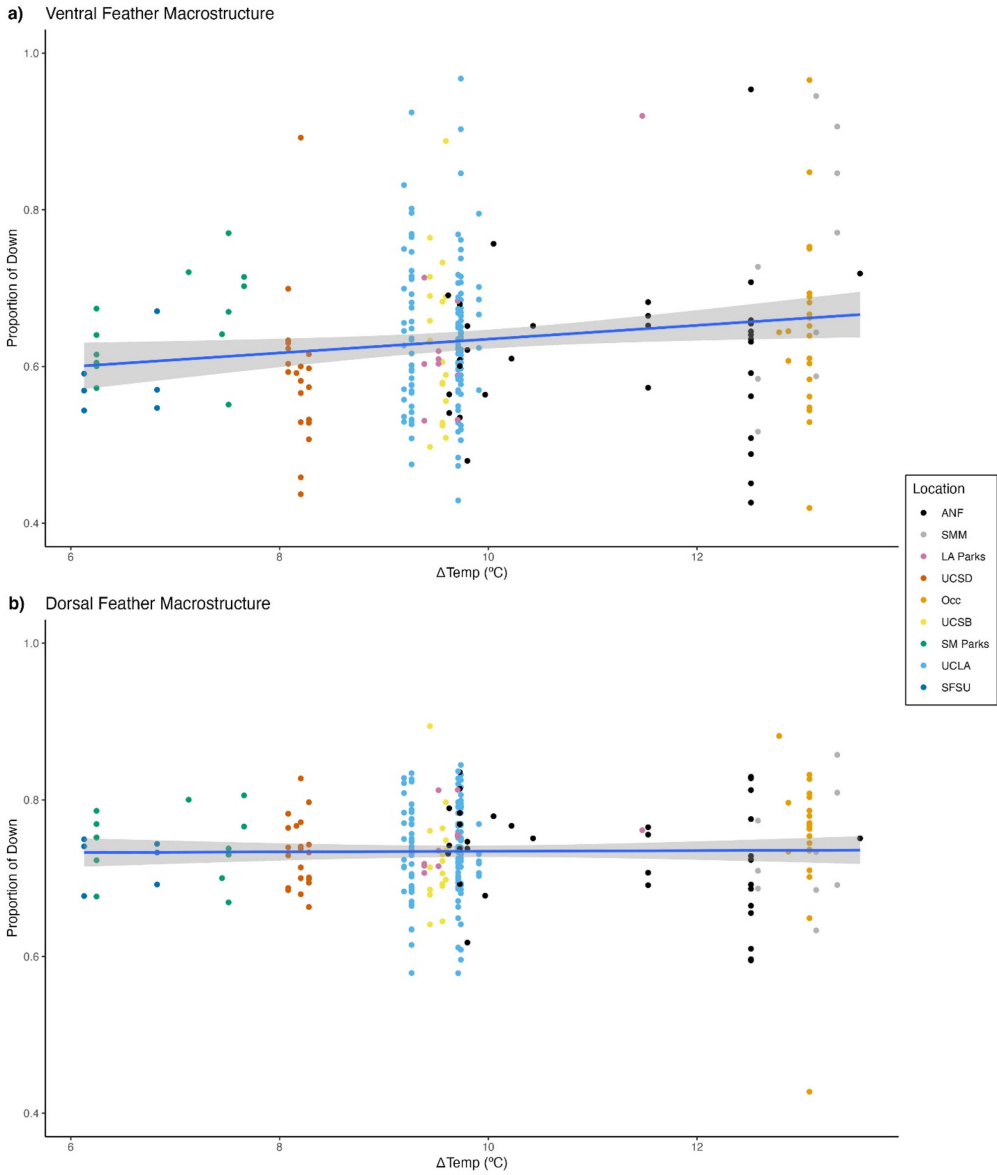
The proportion of down in response to 30-year normal temperature range (ºC) for (**a**) ventral and (**b**) dorsal feathers. Non-urban sites include ANF (black) and SMM (grey). Urban sites include LA Parks (pink), UCSD (dark orange), Occ (light orange), SM Parks (green), UCLA (light blue), SFSU (dark blue). Blue line represents the (**a**) generalized linear model and (**b**) linear model. Shaded grey regions represent the 95% confidence interval.

For ventral feathers, the model with ΔTemp as a predictor variable had the lowest AICc value (Table 1). The proportion of down increased in sites with larger temperature ranges (AICc weight = 0.840, β_1_ = −0.068 ± 0.028, *t* = −2.425, *p* = 0.016; Fig. 4a). The null model (AICc weight = 0.115) and urbanization model (AICc weight = 0.044) were not well supported.

For dorsal feathers, a null model had the lowest AICc scores (AICc weight = 0.953; Table 1), suggesting that neither temperature (AICc weight = 0.027, β_0_ = 0.002 ± 0.002, *t* = 0.864, *p* = 0.389; Fig. 4b) nor urbanization (AICc weight = 0.020, β_0_ = -0.012 ± 0.037, *t* = -0.319, *p* = 0.75) were well supported as predictors of variation in dorsal feather macrostructure.

### Repeatability

Based on a linear model, the number of feathers removed from a given individual did not significantly affect the variance in the proportion of down for that individual (dorsal: β = −0.0014 ± 0.007, t = −0.824, *p* = 0.456; ventral: β = 0.007 ± 0.007, t = 1.079,* p* = 0.341).

## Discussion

Within the context of evolutionary history, urbanization is a recent, but significant source of environmental change^57^. In this study, we analyzed variation in the proportion of down observed in contour feathers collected from Dark-eyed Juncos across an urbanization and temperature gradient in California, USA. Our results showed that urban habitats had more temperature-stable environments. However, neither urbanization nor temperature were correlated with variation in dorsal feathers, and in ventral feathers, temperature was only weakly correlated with an increase in the proportion of down. Contour feathers are essential for several biological functions and have been found to be highly variable, through adaptations and phenotypic plasticity^19,22,25,27,32,35,45^, but our results suggest that rapid environmental changes presented by urbanization do not correspond with rapid morphological variation in feathers in this species.

Cities are known to produce urban-heat islands, but small, localized patches of green space can create climate refugia that obscure or dilute detrimental heat effects on wildlife^1–3,18^. The presence of climate refugia has been reported in California, USA, specifically across the Los Angeles Basin^58,59^ where our study takes place. We report a similar trend, where urbanization does not necessarily correlate with higher ambient temperatures but does result in more stable temperatures. Temperature stability, referring to the difference between the minimum and maximum temperatures, is one aspect of the UHI effect and more biologically relevant for feather macrostructure at this scale^60^. Therefore, we assess how UHI-related temperature stability correlates with differences in feather morphology.

We examined whether the bird’s body region corresponded with a difference in contour feather morphology. We found a large effect of body region, with the proportion of down being significantly higher in dorsal feathers compared to ventral feathers (Fig. 3). This difference is likely the product of two major functions provided by contour feathers: protection from solar radiation^29^ and thermoregulation^20^. Protection from UV radiation increases with a larger pennaceous region and a smaller proportion of down^27,61^, which suggests the Dark-eyed Junco dorsal feathers are providing less UV protection. However, as the dorsal feathers also have more melanin, albeit pheomelanin^62^, this trade-off may be lessened. The ventral feathers, in contrast, have reduced direct exposure to UV light, minimal, if any, melanin pigmentation in the pennaceous barbs^62^, and have a smaller proportion of down. Collectively, these traits suggest that ventral feathers absorb less solar radiation while also retaining less heat^19^. While this explanation, and our study as a whole, primarily focuses on thermoregulation, it is possible that other explanations, including functions related to incubation or water repellence^22,27,32^, may be equally as viable explanations for the observed differences. Future studies could also expand on this observation to assess if there are any physiological effects related to variation in feather morphology on an individual bird and how these differences may constrain evolvability.

Despite urbanization being a major source of habitat changes, we found little support for an effect on contour feather morphology (Table 1). While urban Dark-eyed Junco populations have demonstrated many instances of rapid variation, especially in their behaviors^6,8,40^ and tail feather coloration^38^, this is not a universal occurrence. Some traits, including additional behaviors, remain unchanged^11^, while others, notably body metrics^16^ and even recent studies on tail feather coloration^17^, demonstrate nuanced variation. Therefore, it is not entirely surprising to see that contour feather morphology remained constant across this urban gradient. Contour feathers contribute several functions that are vital for avian biology^27,32^ and may only show variation under very strong selection pressures. Even in cases where contour feathers demonstrate variation in response to urbanization^13,14,25,63^, these may be linked to plasticity caused by dietary changes^25^ that are more prominent in juveniles than adults^13,14^. It is also possible that by sampling shortly after many individuals underwent an annual molt (February – March), we did not capture differences that arise over time as a result of wear^64^. Our study, like other studies on Dark-eyed Juncos^34^, showed that contour feather morphology remains relatively constant. Whether this trend occurs in other bird species, especially urban birds with specialized dietary needs, would be an important area for future study.

Broad climatic conditions correspond with feather morphology variation^19,20,35^, and we were interested in examining whether this was true for a more recent, fine-scale temperature variation. Our results report a weak correlation between the urban heat island effect and the proportion of down in ventral feathers. Based on a generalized linear model, the proportion of down in ventral feathers was lowest in stable climates and increased with larger temperature ranges, suggesting birds in more variable habitats could be increasing heat retention^26,36,45^. This weak correlation could be reflective of the relatively mild temperature gradient examined in our study but our results were consistent with those seen in a study on contour feathers of Dark-eyed Juncos across an elevational gradient^34^. Therefore, it is possible that despite experiencing the UHI effect, this fine-scale temperature variation does not represent a strong selective force on the morphology of contour feathers of adult Dark-eyed Juncos. Although broad climatic conditions appear to be more directly correlated with feather morphology^19,20,35^, future studies could examine contour feather morphology and thermoregulatory behaviors, such as ptiloerection or shivering^24^, to determine if the UHI effect correlates with a more multifaceted plastic response.

Urbanization is a complex phenomenon and our study focuses on the variation in one trait, the proportion of down of contour feathers, across multiple populations. Although we did not find a significant difference, future work is needed to fully assess the nuanced effects of urbanization on wildlife. In addition to the suggestions we have provided, future studies could expand on our results by increasing the geographic range, assessing other aspects of feather morphology, incorporating seasonality which includes molting and breeding, examining the effects of temperature extremes, and potentially including behavioral traits. As urbanization continues to expand and alter local habitats, understanding which traits show rapid variation, and the implication of this variation, will help us understand the impacts of human activity on wildlife.

## Supplementary Information

Supplementary Information.

## Data Availability

Data can be found online on 10.5061/dryad.1jwstqk3t or by contacting the primary corresponding author, Dr. Pamela Yeh (pamelayeh@ucla.edu) or Wilmer Amaya-Mejia (amayamejiaws@gmail.com).
